# Correlation between tuberculin skin test and IGRAs with risk factors for the spread of infection in close contacts with sputum smear positive in pulmonary tuberculosis

**DOI:** 10.1186/1471-2334-14-258

**Published:** 2014-05-13

**Authors:** Maria Luiza de Souza-Galvão, Irene Latorre, Neus Altet-Gómez, María Ángeles Jiménez-Fuentes, Celia Milà, Jordi Solsona, Maria Asunción Seminario, Adela Cantos, Juan Ruiz-Manzano, José Domínguez

**Affiliations:** 1 Unitat de Tuberculosi de Drassanes, Hospital Universitari Vall d’Hebron, Barcelona, Spain; 2 IDIAP Jordi Gol Research Foundation, Barcelona, Spain; 3 Universitat Autònoma of Barcelona, Barcelona, Spain; 4 Servei de Microbiologia, Institut d’Investigació Germans Trias i Pujol, Badalona, Spain; 5 Servei de Pneumologia, Hospital Universitari Germans Trias i Pujol, Badalona, Spain; 6 CIBER Enfermedades Respiratorias, Madrid, Spain

**Keywords:** Tuberculosis infection, Tuberculin skin test, Interferon gamma release assays, IGRA, Overcrowding, Diagnostic delay, Cough

## Abstract

**Background:**

The aim of the study was to assess the correlation between the tuberculin skin test (TST) and *in vitro* interferon-gamma released assays (IGRAs) with risk factors for the spread of infection in smear positive pulmonary tuberculosis (TB) contacts.

**Methods:**

We recruited prospective contacts with smear positive pulmonary TB cases. We looked at human immunodeficiency virus (HIV) infection and other conditions of immunosuppression, presence of BCG vaccination and the degree of exposure to the index case. Patients underwent the TST, chest radiography, sputum analysis when necessary, and IGRA assays (QFN-G-IT and T-SPOT.TB). Presence of cough, diagnostic delay (days between first symptoms and TB diagnostic), contact conditions: room size (square meters) and index of overcrowding (square meters per person) were investigated in the index case.

**Results:**

156 contacts (119 adults, 37 children) of 66 TB patients were enrolled, 2.4 (1-14) contacts per TB case. The positivity of the TST did not correlate with the risk factors studied: presence of cough (p = 0.929); delayed diagnosis (p = 0.244); room size (p = 0.462); overcrowding (p = 0.800). Both QFN-G-IT and T-SPOT.TB, showed significant association with cough (p = 0.001, and p = 0.007) and room size (p = 0.020, and p = 0.023), respectively.

**Conclusions:**

Both IGRA associated better than TST with certain host-related risk factors involved in the transmission of disease, such as the presence of cough.

## Background

The World Health Organization estimates approximately 8.6 million new cases of tuberculosis (TB) annually [1]. Studies published in the 80s showed that 5-10% of recently infected contacts develop active disease within the subsequent 2-5 years after exposure to an infectious source; while another 5-10% percent develop TB sometime during the rest of their lives [2]. In Barcelona, a low TB burden city (incidence: 24.9/100,000 in 2011), a study showed that 25.5% of new TB cases were due to recent transmission [3].

TB prevention relies on the targeted identification and preventive treatment of individuals who carry an increased risk of developing TB [4]. Identification of individuals from these groups, showing the highest risk of developing TB is performed by the evaluation of the presence of an adaptive immunity against *Mycobacterium tuberculosis,* in the absence of active disease. For many decades the tuberculin skin test (TST) has been the immunodiagnostic method of choice for this risk analysis. In the last years, interferon–γ (IFN-γ) release assays (IGRAs), the Quantiferon-TB Gold in Tube (QFN-G-IT) (QIAGEN, Düsseldorf, Germany), and the T-SPOT.TB (Oxford Immunotec Limited, Abingdon, UK), have been introduced into clinical practice as laboratory methods for the immune-diagnosis of latent TB infection (LTBI) [5].

The spread of TB in confined spaces is strongly influenced by the number of infected droplet nuclei present in the air, the viability of the *M. tuberculosis* bacilli and the duration of exposure [6]. Living in a crowded household increases the risk of TB infection [7]. The presence of cough decreases the delay in TB diagnostic, improving suspicion, also increasing the spread of the disease [8]. While several studies have compared the frequency of positive TST and IGRA results in relation to the exposure time to case index [9,10], no study comparing the performance of the TST and IGRAs was targeted directly in living conditions and on overcrowding, as risk factors for the development of LTBI in contacts.

The aim of this study was to assess the association between the positivity of TST and IGRAs with some risk factors for the spread of the disease as: presence of a cough and diagnostic delay in the index case; and the size of the room and the overcrowding in the environment where the contact took place. Furthermore, we compared the performance of the 3 tests, the agreement between them as well as their correlation with the time of exposure.

## Methods

### Study design

We prospectively recruited people who came to the TB Unit in Drassanes (Barcelona, Spain) in our routine contact investigation tasks. The Ethics Committee of the “IDIAP Jordi Gol Research Foundation” provided ethics approval for this study. Patients and parents of children were asked to sign a consent form. Detailed questionnaires were completed with data about prior contact or any prior TST result; data about HIV infection or other causes of immunosuppression were collected and we looked for the presence of BCG scars. We tested using TST and whenever possible, also with QFN-GIT and with T-SPOT.TB. We drew blood only from untreated patients or from patients receiving LTBI treatment for less than 30 days, in order to avoid the effects of the therapy skewing IGRA results [11]. Chest x-Ray and sputum analysis were also done if necessary.

Contacts were eligible if they were exposed to a pulmonary TB sputum smear positive patient index, and excluded if they had active TB, were diagnosed with HIV infection, were pregnant, or if the result of the culture of the patient index was not confirmed to be *M. tuberculosis*. Contacts of sputum smear positive patients were from two different origins: familial (relatives or friends referred to the Unit for TB screening) and community (colleagues or students referred to the Unit for screening with chest x-Ray due to a positive TST). They were also classified according to degree of exposure to the case index in 2 groups: 6 or more hours daily and less than 6 hours daily. Clinicians were blind to IGRA results and microbiologists did not know the clinical history of the patients.

### Clinical and environmental risk factors for TB infection

In the index case we checked for the presence or absence of a cough and considered the days of diagnostic delay, defined as days between the first symptoms and TB diagnostic. As far as the environmental contact conditions, we looked for data on the room size (in square meters) and we used the Floor Area per Person Index. The room size was classified according to the Statistical Institute of Catalonia, slightly adapted to the characteristics of the neighbourhood of our study population. This indicator of Sustainable Development was used by the United Nations, to measure the adequacy of living space, not taking into account cultural differences, as an indicator of overcrowding. We considered overcrowding when the index was less than 15 square meters per person [12]. The data about the index case were obtained by direct interview when the patient was diagnosed in our institution or collected from the database of the TB Programs of Barcelona and Catalonia.

### Tuberculin skin testing

TST was performed by the Mantoux method using two tuberculin units of PPD RT23 (Statens Serum Institut, Copenhagen, Denmark) and read within 48-72 h, using the ball-point pen and ruler method by trained nurses and doctors. All TST ≥ 5 mm were classified as positive result regardless of the BCG defined by the Spanish Pneumology and Thoracic Surgery Society (SEPAR) guidelines [13,14].

### QFN-gold in tube

One ml of blood was drawn per each of the 3 tubes with antigens ready for incubation: (Nil, TB antigens and mitogen). Samples were incubated with stimulatory antigens for 16 h-24 h and the test was performed according to the manufacturer’s instructions. The cut-off value for a positive test was IFN-γ of at least 0.35 IU/mL in the sample after stimulation with the specific antigens, regardless of the result of the mitogen control. The result of the test was considered indeterminate if the antigen-stimulated sample was negative and if the value of the positive control was less than 0.5 IU/mL after subtraction of the value of the nil control, and/or if the nil control was higher than 8.0 IU/ml.

### T-SPOT.TB

The test was performed following manufacturer’s recommendations. 8 ml of blood were drawn from each subject by venipuncture for the isolation of peripheral blood mononuclear cells (PBMCs) in CPT tube (Beckton Dickinson Diagnostics, Franklin Lakes, NJ). The isolated PBMCs were washed twice by centrifugation with RPMI medium (Invitrogen, Auckland, N.Z.), and later resuspended in AIM-V medium (Invitrogen, Auckland, N.Z.). Finally, viable cells were counted with an inverted microscope using the tripan blue method.

After that, the PBMCs were stimulated in each well by medium alone (as nil control), phytohaemagglutinin (as positive control), different peptide panels encompassing the antigens ESAT-6 and CFP-10. Spots were scored using an automated AID ELISPOT plate reader (AID Systems, Strasberg, Germany) and also by naked eye. Test wells were scored as positive if they contained at least six spot-forming cells more than the nil control well, and if this number was at least twice the number of the nil control well. The result of the test was considered undetermined if the antigen-stimulated sample was negative and if the value of the positive control was less than 20 spots, and/or if the number of SFC in the negative control was greater than 10. All blood samples for both tests were processed within 4 h of phlebotomy in the Microbiology Department of the Hospital Universitari Germans Trias i Pujol in Badalona (Spain).

### Statistical analysis

The qualitative variables description is based on the calculation of the number and its percentage, and for quantitative variables, based on calculation of the mean and the standard deviation (SD). The chi-squared test and two-tailed Fisher’s exact test were used to compare qualitative variables. Non-parametric tests (Mann–Whitney, Kolmogorov-Smirnov, Kruskal-Wallis) were used to compare quantitative variables according to the categories of the group of variable. Cohen’s kappa coefficient (k) was used to analyse the concordance and its p value. All analyses were made with SPSS statistical software for Windows (SPSS version 15.0; SPSS, Chigago, IL).

## Results

### Demography

**Figure 1:**
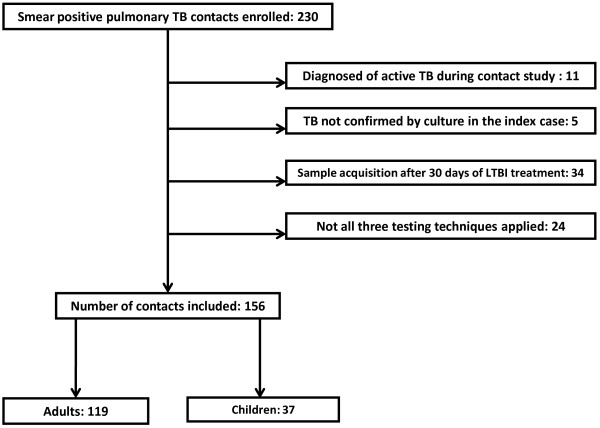
Patients included and causes for exclusion from the study (TB: tuberculosis. LTBI: latent tuberculosis infection).

Table 1 summarizes the demographic and clinical details. 230 contact patients with smear positive TB were recruited for the study. 11 patients were excluded by diagnosis of active TB; 5 by absence of growth of *M. tuberculosis* in the culture sputum of the index case and 34 because the blood samples were collected more than 30 days after they had started treatment of TB infection. 24 contacts did not have all three testing techniques applied. In the end, a total of 156 contacts (119 adults and 37 children) from 66 cases of pulmonary TB sputum smear positive were enrolled (Figure 1). The average number of close contacts per source case was 2.4 and varied from 1 to 14 individuals.

**Table 1:** Characteristics of the study population (n = 156)

**Characteristics**	**Adults n = 119**	**Children n = 37**
**Mean age (SD)**	29.3 (10.5)	9.2 (2.4)
**Gender**		
Female	67 (56.3)	21 (56.8)
Male	52 (43.7)	16 (43.2)
**BCG-vaccinated**		
Yes	69 (58)	25 (67.6)
No	50 (42)	12 (32.4)
**Place of birth**		
Spain	60 (50.4)	12 (32.4)
Outside Spain	59 (49.6)	25 (67.6)
America	42 (71.2)	19 (76)
Eastern Europe Mediterranean	7 (11.9)	1 (4)
Europe	6 (5)	5 (13.5)
South Asia	1 (1.7)	0 (0)
Western Pacific	3 (5.1)	0 (0)
**Type of contact**		
Familial	45 (37.8)	25 (67.6)
Community	74 (62.1)	12 (32.4)
**Degree of exposure**		
≥ 6 hours daily	46 (38.6)	16 (43.2)
< 6 hours daily	73 (61.4)	21 (56.8)
**Cough in index case**		
Yes	37 (52.1)	23 (92)
No	34 (47.9)	2 (8)

Data are presented as n (%) or means ± SD. *BCG*: Bacille Calmette-Guérin.

### Agreement between tests

Overall agreement of TST and both IGRAs was mostly slight (Table 2). Nevertheless, in BCG-unvaccinated children reached the best agreement. The overall agreement between both IGRAs was substantial; with no differences in the agreement in BCG-vaccinated and non-BCG-vaccinated taking into account children and adults together.

**Table 2:** Agreement between tests and Κ coefficients

**Patient characteristics**	**TST *versus *** **T-SPOT-TB**		**TST *versus *** **QFN-IT**		**T.SPOT-TB *versus *** **QFN-IT**	
	**Agreement (%)**	**Kappa**	**Agreement (%)**	**Kappa**	**Agreement (%)**	**Kappa**
**Total**						
All	62.6	0.172	50.91	0.121	82.82	0.659
BCG	51.58	0.119	42	0.078	83.84	0.664
No BCG	79.37	0.310	64.62	0.224	81.25	0.604
**Adults**						
All	58.97	0.148	45.6	0.100	81.45	0.629
BCG	47.83	0.101	34.72	0.056	80.82	0.580
No BCG	75	0.256	60.38	0.195	82.35	0.637
**Children**						
All	75	0.288	69.23	0.235	86.84	0.721
BCG	64	0.199	62.96	0.182	92	0.838
No BCG	93.33	0.634	83.33	0.429	76.92	0.418

*BCG*: Bacille Calmette-Guérin. *TST*: tuberculin skin test. *QFN-G-IT*: Quantiferon-TB Gold in tube.

### Positive test results for all techniques

**Figure 2:**
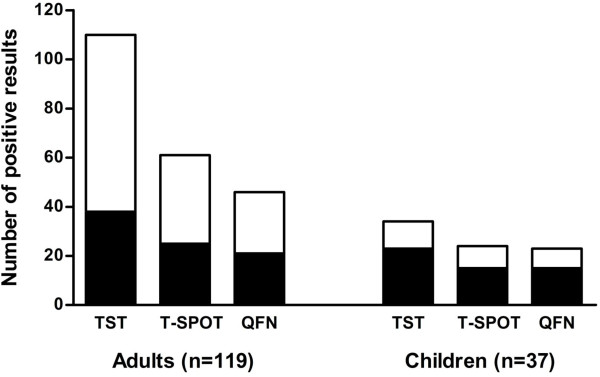
Number of positive results obtained by TST and both IGRA tests (T-SPOT.TB and QFN-G-IT) in adults and children, including information in each case of the origin of the contact: familial (■) or from the community (□).

The results of TST and IGRAs are summarised separately into adults and children according with the origin of the contact (familial or community contact) in Figure 2, and also according to the presence or absence of BCG vaccine and the degree of exposure (Tables 3 and 4).

**Table 3:** Results of interferon-γ release assays and tuberculin skin test in adults and children BCG-unvaccinated

		**Adults**				**Children**			
**Technique**		**Degree of exposure**				**Degree of exposure**			
		**> 6 h daily**	**< 6 h daily**	**Total**	**p**	**> 6 h daily**	**< 6 h daily**	**Total**	**p**
**TST**	Positive	18 (94.7)	28 (90.3)	46 (92)	0.820	2 (100)	9 (90)	11 (91)	0.999
	Negative	1 (5.3)	3 (9.7)	4 (8)		0 (0)	1 (10)	1 (9)	
**QFN-G-IT**	Positive	13 (68.4)	14 (45.2)	27 (54)	0.173	2 (100)	7 (70)	9 (75)	0.999
	Negative	6 (31.6)	17 (54.8)	23 (46)		0 (0)	3 (30)	3 (25)	
	Indeterminate	0 (0)	0 (0)	0 (0)		0 (0)	0 (0)	0 (0)	
**T.SPOT-TB**	Positive	15 (78.9)	18 (58)	33 (66)	0.223	2 (100)	8 (80)	10 (83.3)	0.999
	Negative	4 (21.1)	11 (35.5)	15 (30)		0 (0)	2 (20)	2 (16.7)	
	Indeterminate	0 (0)	2 (6.5)	2 (4)		0 (0)	0 (0)	0 (0)	

Data are presented as n (%). *BCG*: Bacille Calmette-Guérin. *TST*: tuberculin skin test. *QFN-G-IT*: Quantiferon-TB Gold in tube.

Two-tailed statistical test was used to compare the qualitative results of TST, QFN-G-IT and T-SPOT.TB, respectively, between the two degree of exposure categories in adults and children.

**Table 4:** Results of interferon-γ release assays and tuberculin skin test in adults and children BCG-vaccinated

		**Adults**				**Children**			
**Technique**		**Degree of exposure**				**Degree of exposure**			
		**> 6 h daily**	**< 6 h daily**	**Total**	**p**	**> 6 h daily**	**< 6 h daily**	**Total**	**p**
**TST**	Positive	27 (100)	37 (88.1)	64 (92.7)	0.998	13 (92.9)	10 (90.9)	23 (92)	0.859
	Negative	0 (0)	5 (11.9)	5 (7.3)		1 (7.1)	1 (9.1)	2 (8)	
**QFN-G-IT**	Positive	9 (33.3)	10 (23.8)	19 (27.5)	0.622	9 (64.3)	5 (45.5)	14 (56)	0.350
	Negative	17 (63)	32 (76.2)	49 (71)		5 (35.7)	6 (54.5)	11 (44)	
	Indeterminate	1 (3.7)	0 (0)	1 (1.5)		0 (0)	0 (0)	0 (0)	
**T.SPOT-TB**	Positive	14 (51.8)	14 (33.3)	28 (40.6)	0.175	9 (64.3)	5 (45.5)	14 (56)	0.350
	Negative	13 (48.2)	28 (66.7)	41 (59.4)		5 (35.7)	6 (54.5)	11 (44)	
	Indeterminate	0 (0)	0 (0)	0 (0)		0 (0)	0 (0)	0 (0)	

Data are presented as n (%). BCG: Bacille Calmette-Guérin. TST: tuberculin skin test. QFN-G-IT: Quantiferon-TB Gold in tube.

Two-tailed statistical test was used to compare the qualitative results of TST, QFN-G-IT and T-SPOT.TB, respectively, between the two degree of exposure categories in adults and children.

#### BCG-unvaccinated

The results of the TST showed 46 (92%) positive adults and 11 (91%) positive children (Table 3). The QFN-G-IT was positive in 27 (54%) adults and 9 (75%) children, while the T-SPOT.TB was positive in 33 (66%) and indeterminate in 2 (4%) adults; and positive in 10 (83.3%) children with no indeterminate results.

#### BCG-vaccinated

Percentages of positive results in TST were similar to those of non-vaccinated individuals (92.8% and 92% in adults and children, respectively) (Table 4). In contrast, IGRAs detected less positive results compared to the unvaccinated groups, except for T-SPOT.TB in children.

### Positive test results according to degree of exposure

#### BCG-unvaccinated contacts

TST positive results did not correlate with the degree of exposure in adults, showing identical percentage of positive results independently of the degree exposure (Table 3). In contrast, although IGRAs showed a higher amount of positive results with the increase of hours of exposure to the index case, the differences did not reached statistical significance.

Taking into account the same degree of exposure, significant differences were found between the positive results using the 3 techniques. TST compared with QFN-G-IT and T-SPOT.TB showed more significant positive results in both degrees of exposure: more than 6 hours (p = 0.007 and p = 0.022, respectively) and less than 6 hours (p < 0.001, respectively) in adults. The low number of BCG-unvaccinated children included, did not allow us to establish if there was a statistical association in this group.

#### BCG-vaccinated contacts

TST did not show a significant difference in the number of positive results according to the degree of exposure, neither in adults nor in children (Table 4). In opposite, although IGRAs showed a higher amount of positive results with the increase of hours of exposure to the index case, the differences did not reached statistical significance.

Taking into account the same degree of exposure, significant differences were found between the positive results using the 3 techniques. TST compared with QFN-G-IT and T-SPOT.TB showed more positive results in both degrees of exposure: more than 6 hours (p < 0.001, respectively) and less than 6 hours (p < 0.001, respectively) in adults and the same in children (p = 0.018 and 0.016, respectively).

### Quantitative evaluation of T-cell response according to the degree of exposure

**Figure 3:**
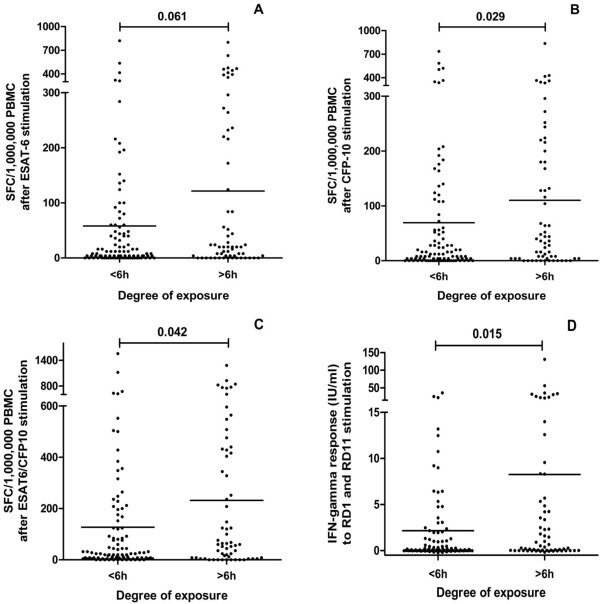
Number of responder T cells to ESAT-6 (A), CFP-10 (B), ESAT6/CFP10 (C), and IFN-γ released after specific antigens stimulation (D) in more and less than 6 hours of exposition to the index case.

We studied T-cell quantitative enumeration after stimulation with ESAT-6, CFP-10 and also the sum of both antigens together (ESAT6/CFP10) with T-SPOT.TB; and the amount of IFN-γ release in QFT-G-IT, in the contacts included, according to the different degrees of exposure to the index case. In adults, we found a higher amount of responding T cells after stimulation with CFP-10 (p = 0.029), ESAT6/CFP10 (p = 0.042) and in the amount of IFN-γ released (p = 0.015) (Figure 3) in contacts with >6 h of exposure, in comparison to the contacts with <6 h of exposure. In children, this analysis was not possible, given the low number of children included.

### Positive results according to clinical and environmental risk factors

We studied 4 risk factors likely associated with TB infection: “presence of cough”, “days of diagnostic delay”, “size of the household contact” and “overcrowding”, and their relation with the positivity of the TST, QFN-GIT and T-SPOT.TB. In the paediatric population, the positivity of the techniques was not correlated with any of the risk factors studied. Data concerning the adult population is presented in Table 5. For the TST, the positivity of the test was not correlated with any of the risk factors studied. On the other hand, we found that the positivity of both IGRAs tests were associated with presence of cough and with the household size, but in this case, inversely to what was expected: we found more positive results, the more square meters the room contained. In T-SPOT.TB results, there was a trend towards an association with diagnostic delay. Overcrowding was not associated with the positivity of any of the tests. The unexpected result of the household size was attributed to the association found between household size and the degree of exposure: contacts living in larger homes had been exposed longer to the index case. Logistic regression analysis was not performed since the number of cases having all the variables to be analyzed, was insufficient.

**Table 5:** Risk factors associated with the positivity of the TST, QFN-G-IT and T-SPOT.TB in adults

**Risk factors**	**TST**			**QFN-G-IT**			**T-SPOT.TB**		
	**n (%)**			**n (%)**			**n (%)**		
	**Positive**	**Negative**	**p**	**Positive**	**Negative**	**p**	**Positive**	**Negative**	**p**
**Cough**									
Yes	34 (94.4)	2 (5.6)	0.929	22 (61.1)	14 (38.9)	0.001	25 (69.5)	11 (30.5)	0.007
Not	31 (93.9)	2 (6.1)		7 (21.2)	26 (78.8)		12 (36.4)	21 (63.6)	
**Diagnostic delay**									
<30	35 (97.2)	1 (2.8)	0.244	11 (30.5)	25 (69.5)	0.565	14 (38.9)	22 (61.1)	0.077
30-59	13 (76.5)	4 (23.5)		7 (41.2)	10 (58.8)		7 (41.2)	10 (58.8)	
60-89	17 (85)	3 (15)		7 (35)	13 (65)		9 (45)	11 (55)	
>89	25 (100)	0 (0)		12 (48)	13 (52)		18 (72)	7 (28)	
**Room’s size (m**^**2**^**)**									
≤50	2 (66.7)	1 (33.3)	0.462	1 (33.3)	2 (66.7)	0.020	2 (66.7)	1 (33.3)	0.023
51-79	15 (78.9)	4 (21.1)		4 (21.1)	15 (78.9)		6 (31.6)	13 (68.4)	
≥80	19 (90.5)	2 (9.5)		14 (66.7)	7 (33.3)		16 (76.2)	5 (23.8)	
**Overcrowding**									
≤15	7 (87.5)	1 (12.5)	0.800	2 (28.6)	5 (71.4)	0.480	5 (62.5)	3 (37.5)	0.820
>15	26 (83.9)	5 (16.1)		16 (51.6)	15 (48.4)		18 (58.1)	13 (41.9)	

TST: tuberculin skin test. QFN-G-IT: Quantiferon-TB Gold in tube.

Diagnostic delay: days between first symptoms and TB diagnostic. Overcrowding: floor area index per person (square meters per person).

Two-tailed statistical test was used to compare the qualitative results of TST, QFN-G-IT and T-SPOT.TB, respectively, between the different categories of the risk factors studied.

## Discussion

To our knowledge, this is the first study that has investigated the role of the environment and of host-related risk factors for TB infection using TST and IGRAs. We compared the risk of positivity of TST, QFN-GIT and T-SPOT.TB and related them to risk factors of the index case, such as presence of a cough and delayed diagnosis, and also included environmental characteristics, such as household size and overcrowding.

The use of IGRAs has increased specificity in the diagnosis of LTBI, and has proven greater cost efficiency, especially in the use of preventive therapy [15,16]. Several studies have compared the performance of the IGRAs against the performance of TST, taking into account the time of exposure from a contagious source and the infectiousness of the source [17-19].

In our experience, the agreement between IGRA tests and TST was slight (especially in BCG-vaccinated), while the agreement between both IGRA tests was substantial and similar in BCG-unvaccinated and BCG-vaccinated contacts. The results suggest, as in previous studies, that IGRA tests are less affected by BCG vaccination than TST [9,19].

In general there is a recognized low capacity of IGRAs to detect LTBI in children, especially in young children. In our study, it is important to point out that the percentage of TST-positive results (in children and adults) was high because most were referred to the Unit for study by positive TST after a contact. This may explain the high percentage of positive results obtained in children included in the study.

A possible explanation for the lack of relation observed between the positivity of the tests and the degree of exposure, could be the fact that we enrolled only contacts of patients showing smear-positive TB. This may have biased the sample of patients towards being more advanced in the disease, thus underestimating the effects of other variables. This inclusion criteria, was used in order to increase the likelihood of positive TST, attempting to get the majority of cases due to recent contact with the case study and not simply due to a previous infection.

The number of responder T cells and the amount of IFN-γ released could be a better indicator of infectiousness, than the qualitative positivity of an IGRA test, as seen in other studies [20]. We even found correlation between the degree of exposure and the number of responder T cells to CFP-10 and ESAT6/CFP10 by TSPOT.TB and the amount of IFN-γ released in adults.

Most transmissions occur between the onset of coughing and the starting of the treatment [21]. It is known that the delay in diagnosis determines disease progression, increasing affected lung areas with the occurrence of cavitated lesions, extending the probability of infecting a new contact. In this study, neither TST nor IGRAs demonstrated to be related to diagnostic delay.

Sputum smear positivity should be considered a risk factor for infectiousness but there are both epidemiologic [22] and experimental studies [23] that have shown considerable variability of infectiousness among sputum smear-positive patients. Some studies suggested that the presence of a cough could generate potentially infectious aerosols that may increase the risk of disease transmission, regardless of the sputum smear status in the index case [24,25]. Our results showed that the positivity of both IGRA tests was associated with the presence of a cough. The potential of infectiousness is related to the patient’s ability to aerosolise bacilli and to the number of bacilli that are aerosolised. In a study in Uganda, contacts of TB patients who produced high aerosols were more likely to have a new infection, as opposed to those who produced low aerosols, and the aerosol negative cases [26].

Since we enrolled only contacts with smear positive TB patients, it was not possible to examine the effect of smear negative and the positivity of tests when the index case had a cough. Guwatudde et al. [27], using TST, studied 1,206 household contacts with smear positive pulmonary. All the TB index cases had a cough for more than 3 weeks and 86% had an advanced disease. Among the host-related risk factors (including overcrowding), they found that only cavitary disease and a prolonged contact with an index case were independently associated with TB in the contact, and the “muzigo” type of housing; a building with multiple rooms that often share air space.

These findings are similar to those of our study, where we did not find a correlation between the overcrowding with the positivity of the tests. The number of patients living in crowded conditions was low in our sample, probably due to the characteristics of the individuals: almost 50% natives and 50% immigrants from Latin American countries, a kind of population with enough resources to live in family groups, in relatively good living conditions. The other type of population was from the community group, usually people sharing the same air for a full working day. In our study, we found a reverse correlation between the size of the room and the positivity of the IGRAs, but associated this correlation with a higher degree of exposure.

Although data about ventilation was not collected in this study, it suggests that in relation to environmental risk factors, more than the number of people in contact with the TB case in a limited environment, it is the ventilation of the room that is the main determinant in the transmission of infection in addition to the time of exposure. A study carried out in Peru found that opening windows and doors provided more than double the amount of ventilation of the mechanical ones and the risk of TB infection was lower [28].

The main limitation of our study was the small number of patients included, given the difficulty of enrolling patients with smear-positive pulmonary TB in our environment. However, the results are sufficiently consistent to show the strong association between the IGRAs and the risk factors involved in the LTBI.

## Conclusions

In conclusion, comparing the performance of the TST and IGRA tests in contacts with smear positive pulmonary TB, we found that both IGRAs were associated with certain host-related risk factors involved in the transmission of disease, such as the presence of cough. On the other hand, the study suggests that some environmental risk factors, such as the lack of ventilation associated with the time of exposure, may be crucial for the transmission, aside from the status smear of the index case and the diagnostic delay. A larger study would be needed, in order to confirm all the findings and better characterize the true role of the different variables involved in the transmission of the disease.

## Abbreviations

TST: Tuberculin skin test; IGRA: Interferon-gamma released assays HIV, Human immunodeficiency virus; TNF: Tumor necrosis factor; QFN-G-IT: Quantiferon-TB Gold in tube; LTBI: Latent tuberculosis infection.

## Competing interests

None of the investigators have relevant financial interest in or a competing interest with the subject matter or materials discussed in this manuscript. None of the Scientific Societies, neither QIAGEN nor Oxford Immunotec (Abingdon, UK) had a hole in the study, conduct, collection, management, analysis, or interpretation of the data, or preparation, review, or approval of the manuscript.

## Authors’ contributions

MLSG, IL and NAG conceived and designed the study. MLSG, NAG, CM, MAJF, JS, MAS, AC and JRM contributed to acquisition and analysis of data from hospital wards, and IL and JD to acquisition and analysis of data in the laboratory. MLSG, IL and JD wrote the manuscript with significant contributions from NAG, MAJF, CM, JS, MAS, AC and JRM. All authors read and approved the final manuscript.

## Authors’ information

Maria Luiza de Souza-Galvão: MD, Irene Latorre: MSc, Ph.D, Neus Altet-Gómez: MD, Ph.D, Celia Milá MD, María Ángeles Jiménez MD, Jordi Solsona MD, Maria Asunción Seminario MD, Adela Cantos BSc, Juan Ruiz-Manzano: MD, Ph.D, and José Domínguez MSc, PhD.

The authors are members of the European Tuberculosis Network (TB-NET) Group.

J. Dominguez is a researcher funded from the *Miguel Servet* programme of the *Instituto de Salud Carlos III* (Spain).

Irene Latorre is currently a research from ICREA Infection Biology Laboratory, Department of Experimental and Health Sciences, Universitat Pompeu Fabra, Barcelona, (Spain).

## Pre-publication history

The pre-publication history for this paper can be accessed here:

http://www.biomedcentral.com/1471-2334/14/258/prepub

## References

[B1] World Health Organization Global tuberculosis report 2013 (WHO/HTM/TB/2013.11) Geneva, Switzerland 2013 http://www.who.int/tb/publications/global_report/en/

[B2] Styblo K Recent advances in epidemiological research in tuberculosis Adv Tuberc Res 1980 14 1 63 7395639

[B3] Borrell S Español M Orcau A Tudó G March F Caylà JA Jansà JM Alcaide F Martín-Casabona N Salvadó M Martínez JA Vidal R Sánchez F Altet N Coll P González-Martín J Factors associated with differences between conventional contact tracing and molecular epidemiology in study of tuberculosis transmission and analysis in the city of Barcelona, Spain J Clin Microbiol 2009 14 198 204 10.1128/JCM.00507-08 19020067 PMC2620856

[B4] Erkens CG Kamphorst M Abubakar I Bothamley GH Chemtob D Haas W Migliori GB Rieder HL Zellweger JP Lange C Tuberculosis contact investigation in low prevalence countries: a European consensus Eur Respir J 2010 14 925 949 10.1183/09031936.00201609 20889463

[B5] Diel R Goletti D Ferrara G Bothamley G Cirillo D Kampmann B Lange C Losi M Markova R Migliori GB Nienhaus A Ruhwald M Wagner D Zellweger JP Huitric E Sandgren A Manissero D Interferon-gamma release assays for the diagnosis of latent mycobacterium tuberculosis infection: a systematic review and meta-analysis Eur Respir J 2011 14 88 99 10.1183/09031936.00115110 21030451

[B6] Beggs CB Noakes CJ Sleigh PA Fletcher LA Siddiqi K The transmission of tuberculosis in confined spaces: an analytical review of alternative epidemiological models Int J Tuberc Lung Dis 2003 14 1015 1026 14598959

[B7] Tornee S Kaewkungwal J Fungladda W Silachamroon U Akarasewi P Sunakorn P The association between environmental factors and tuberculosis infection among household contacts Southeast Asian J Trop Med Public Health 2005 14 Suppl 4 221 224 16438213

[B8] Altet Gómez MN Alcaide Megías J Canela Soler J Milá Augé C Jiménez Fuentes MA de Souza Galvao ML Solsona Peiró J Pulmonary symptomatic tuberculosis’ diagnostic delay study Arch Bronconeumol 2003 14 146 152 12716554 10.1016/s0300-2896(03)75348-4

[B9] Domínguez J Ruiz-Manzano J De Souza-Galvão M Latorre I Milà C Blanco S Jiménez MA Prat C Lacoma A Altet N Ausina V Comparison of two commercially available gamma interferon blood tests for immunodiagnosis of tuberculosis Clin Vaccine Immunol 2008 14 168 171 10.1128/CVI.00364-07 17978008 PMC2223867

[B10] Diel R Loddenkemper R Meywald-Walter K Gottschalk R Nienhaus A Comparative performance of tuberculin skin test, QuantiFERON-TB-gold in tube assay, and T-spot.TB test in contact investigations for tuberculosis Chest 2009 14 1010 1018 10.1378/chest.08-2048 19017873

[B11] Domínguez J De Souza-Galvão M Ruiz-Manzano J Latorre I Prat C Lacoma A Milà C Jiménez MA Blanco S Maldonado J Altet N Ausina V T-cell responses to the *Mycobacterium tuberculosis*-specific antigens in active tuberculosis patients at the beginning, during, and after antituberculosis treatment Diagn Microbiol Infect Dis 2009 14 43 51 10.1016/j.diagmicrobio.2008.09.010 19026511

[B12] UN Department of Economic and Social Affairs. Division for Sustainable Development CSD theme indicator framework from 2001 http://www.un.org/esa/sustdev/csd/csd9_indi_bp3.pdf

[B13] Ruiz-Manzano J Blanquer R Calpe JL Caminero JA Caylà J Domínguez JA García JM Vidal R Spanish Society of Pulmonology and Thoracic Surgery Diagnosis and treatment of tuberculosis Arch Bronconeumol 2008 14 551 566 19006636

[B14] González-Martín J García-García JM Anibarro L Vidal R Esteban J Blanquer R Moreno S Ruiz-Manzano J Consensus document on the diagnosis, treatment and prevention of tuberculosis Arch Bronconeumol 2010 14 255 274 20444533 10.1016/j.arbres.2010.02.010

[B15] Latorre I De Souza-Galvão M Ruiz-Manzano J Lacoma A Prat C Altet N Ausina V Domínguez J Evaluating the non-tuberculous mycobacteria effect in the tuberculosis infection diagnosis Eur Respir J 2010 14 338 342 10.1183/09031936.00196608 20123845

[B16] Nienhaus A Schablon A Costa JT Diel R Systematic review of cost and cost-effectiveness of different TB-screening strategies BMC Health Serv Res 2011 14 247 10.1186/1472-6963-11-247 21961888 PMC3196701

[B17] Shams H Weis SE Klucar P Lalvani A Moonan PK Pogoda JM Ewer K Barnes PF Enzyme-linked immunospot and tuberculin skin testing to detect latent tuberculosis infection Am J Respir Crit Care Med 2005 14 1161 1168 10.1164/rccm.200505-748OC 16081545 PMC2718400

[B18] Lalvani A Pathan AA Durkan H Wilkinson KA Whelan A Deeks JJ Reece WH Latif M Pasvol G Hill AV Enhanced contact tracing and spatial tracking of Mycobacterium tuberculosis infection by enumeration of antigen-specific T cells Lancet 2001 14 2017 2021 10.1016/S0140-6736(00)05115-1 11438135

[B19] Altet-Gómez N De Souza-Galvao M Latorre I Milà C Jiménez MA Solsona J Cantos A Zamora JJ Ruiz-Manzano J Ausina V Domínguez J Diagnosing TB infection in children: analysis of discordances using *in vitro* tests and the tuberculin skin test Eur Respir J 2011 14 1166 1174 10.1183/09031936.00022710 20729220

[B20] Latorre I De Souza-Galvão M Ruiz-Manzano J Lacoma A Prat C Fuenzalida L Altet N Ausina V Domínguez J Quantitative evaluation of T-cell response after specific antigen stimulation in active and latent tuberculosis infection in adults and children Diagn Microbiol Infect Dis 2009 14 236 246 10.1016/j.diagmicrobio.2009.07.015 19822269

[B21] Storla DG Yimer S Bjune GA A systematic review of delay in the diagnosis and treatment of tuberculosis BMC Public Health 2008 14 15 10.1186/1471-2458-8-15 18194573 PMC2265684

[B22] Brooks SM Lassiter NL Young EC A pilot study concerning the infection risk of sputum positive tuberculosis patients on chemotherapy Am Rev Respir Dis 1973 14 799 804 4741874 10.1164/arrd.1973.108.4.799

[B23] Sultan L Nyka W Mills C O’Grady F Wells W Riley RL Tuberculosis disseminators. A study of the variability of aerial infectivity of tuberculous patients Am Rev Respir Dis 1960 14 358 369 13835667 10.1164/arrd.1960.82.3.358

[B24] Fennelly KP Martyny JW Fulton KE Orme IM Cave DM Heifets LB Cough-generated aerosols of mycobacterium tuberculosis: a new method to study infectiousness Am J Respir Crit Care Med 2004 14 604 609 10.1164/rccm.200308-1101OC 14656754

[B25] Fennelly KP Jones-López EC Ayakaka I Kim S Menyha H Kirenga B Muchwa C Joloba M Dryden-Peterson S Reilly N Okwera A Elliott AM Smith PG Mugerwa RD Eisenach KD Ellner JJ Variability of infectious aerosols produced during coughing by patients with pulmonary tuberculosis Am J Respir Crit Care Med 2012 14 450 457 10.1164/rccm.201203-0444OC 22798319 PMC3443801

[B26] Jones-López EC Namugga O Mumbowa F Ssebidandi M Mbabazi O Moine S Mboowa G Fox MP Reilly N Ayakaka I Kim S Okwera A Joloba M Fennelly KP Cough aerosols of mycobacterium tuberculosis predict new infection: a household contact study Am J Respir Crit Care Med 2013 14 1007 1015 10.1164/rccm.201208-1422OC 23306539 PMC3707366

[B27] Guwatudde D Nakakeeto M Jones-Lopez EC Maganda A Chiunda A Mugerwa RD Ellner JJ Bukenya G Whalen CC Tuberculosis in household contacts of infectious cases in Kampala, Uganda Am J Epidemiol 2003 14 887 898 10.1093/aje/kwg227 14585767 PMC2869090

[B28] Escombe AR Oeser CC Gilman RH Navincopa M Ticona E Pan W Martínez C Chacaltana J Rodríguez R Moore DA Friedland JS Evans CA Natural ventilation for the prevention of airborne contagion PLoS Med 2007 14 e68 10.1371/journal.pmed.0040068 17326709 PMC1808096

